# Supplementary figures and images for: PBX3 as a biomarker for the early diagnosis and prediction of prognosis of glioma
Source: PLoS One. 2024 Feb 7;19(2):e0293647. doi: 10.1371/journal.pone.0293647 (PMC10849273; doi:10.1371/journal.pone.0293647)

1 GEPIA http://gepia.cancer-pku.cn/detail.php?gene=&clicktag=boxplot


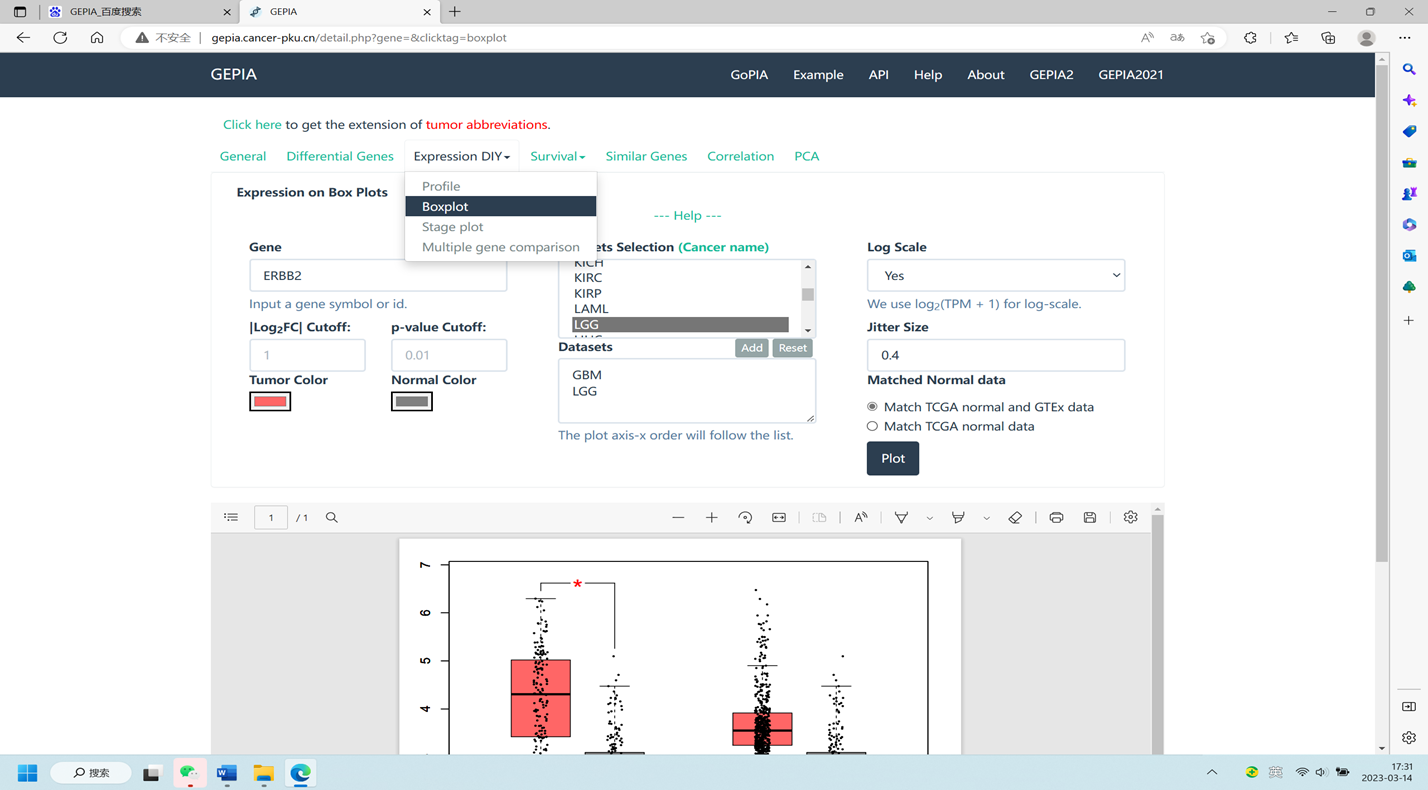

Supplement: S1 Fig — (A-C) Expression level of PBX3 in full-grade gliomas, LGG and GBM in comparison with the normal brain tissues according to TCGA and GTEx. (D) Expression level of PBX3 in LGG and GBM in comparison with the normal brain tissues according to GEPIA. (E) and (F) The expression level of PBX3 between LGG and GBM according to TCGA and CGGA databases. (G) and (H) The expression level of PBX3 in histological types of gliomas according to TCGA and CGGA databases. (I) The expression level of PBX3 in glioma cell lines according to CCLE databases. (ZIP) [file pone.0293647.s001.zip › FIG1/Fig 1D.docx]

1 http://gepia.cancer-pku.cn/detail.php?gene=&clicktag=boxplot


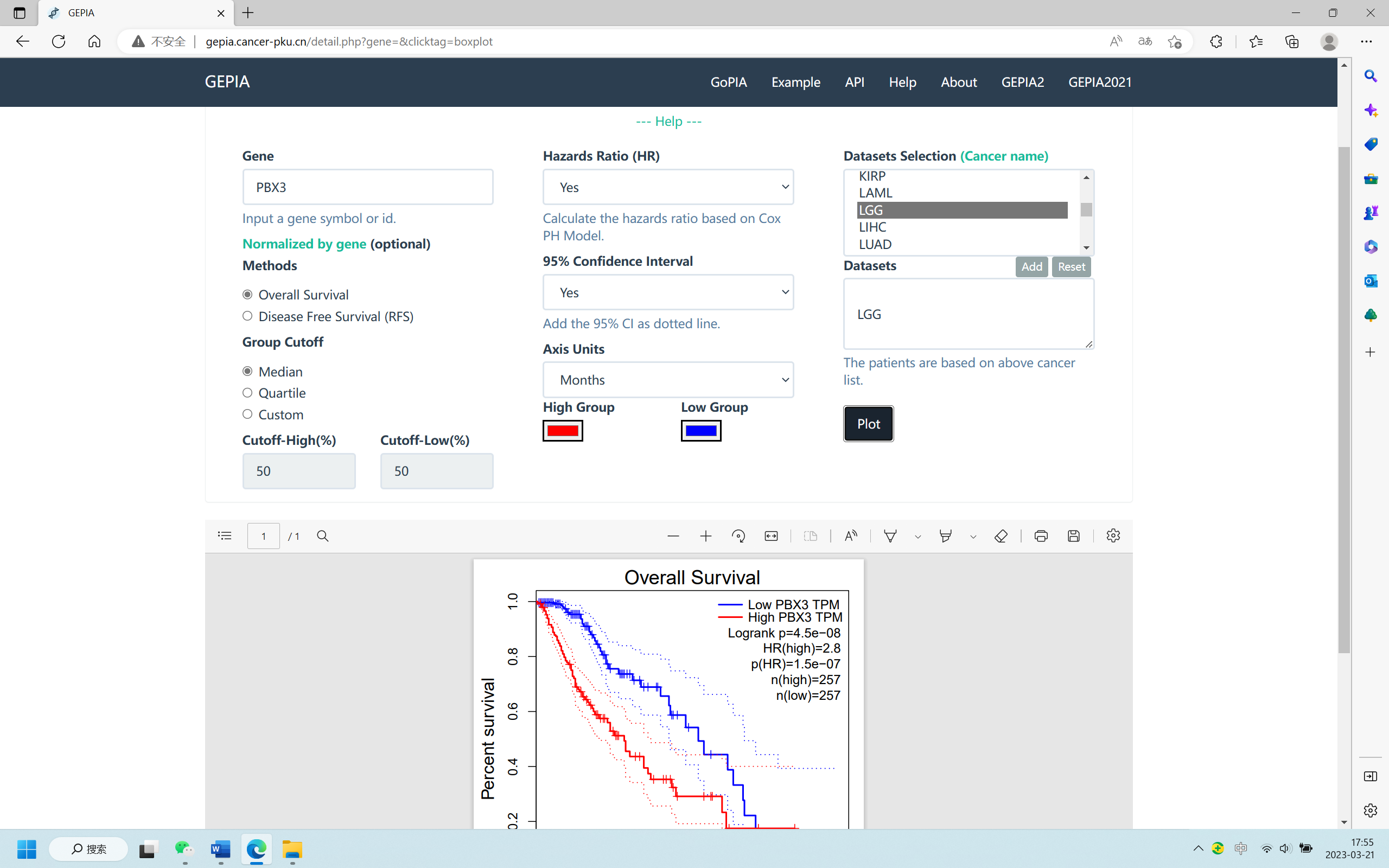


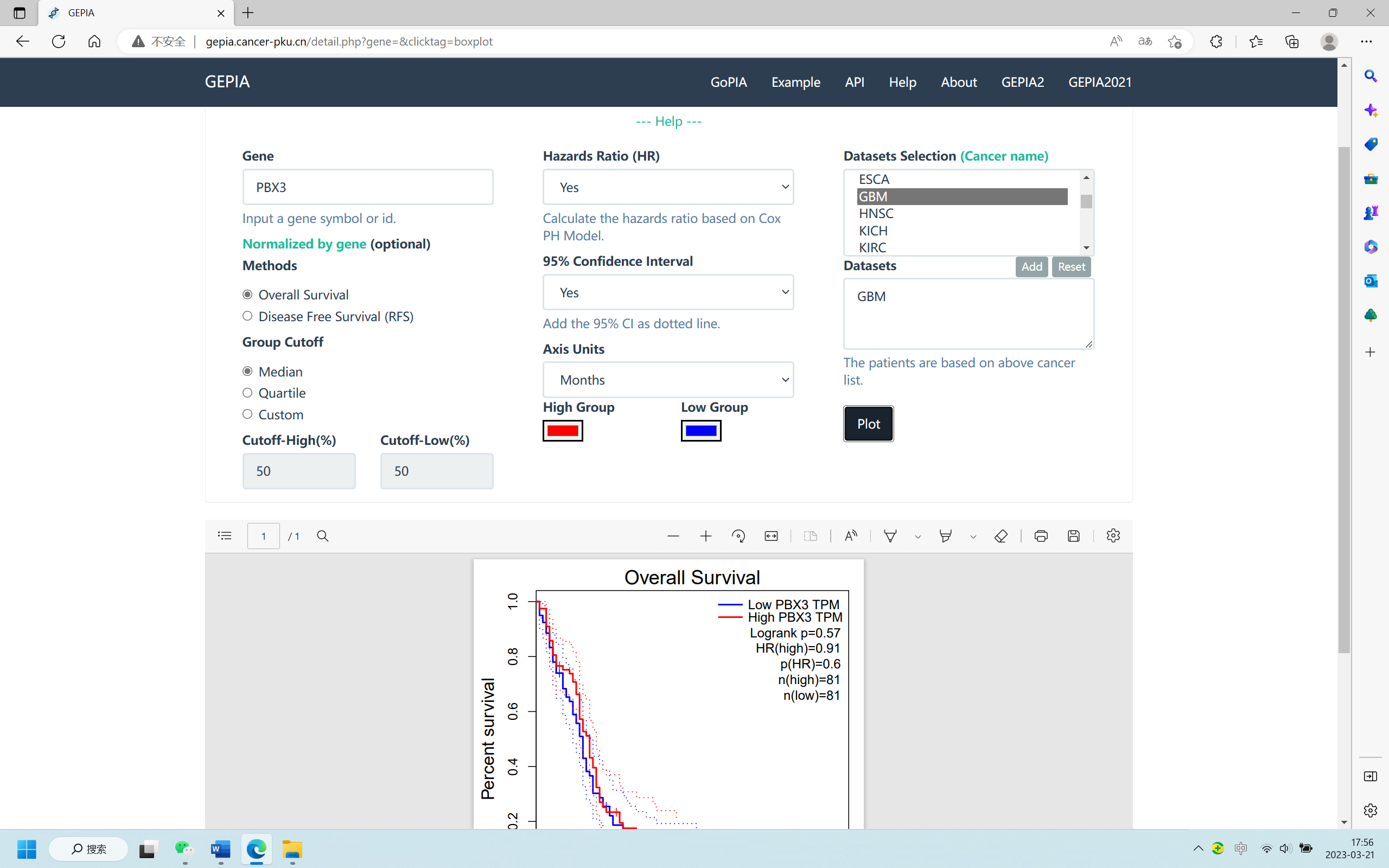

Supplement: S3 Fig — (A and B), full-grade gliomas according to TCGA and CGGA databases;(C-E), LGG according to TCGA, CGGA and GEPIA databases;(F-H), GBM according to TCGA, CGGA and GEPIA databases. (ZIP) [file pone.0293647.s003.zip › Fig 3/Fig3E and Fig3H.docx]

1 GEPIA http://gepia.cancer-pku.cn/detail.php?gene=&clicktag=boxplot

Fig 4C


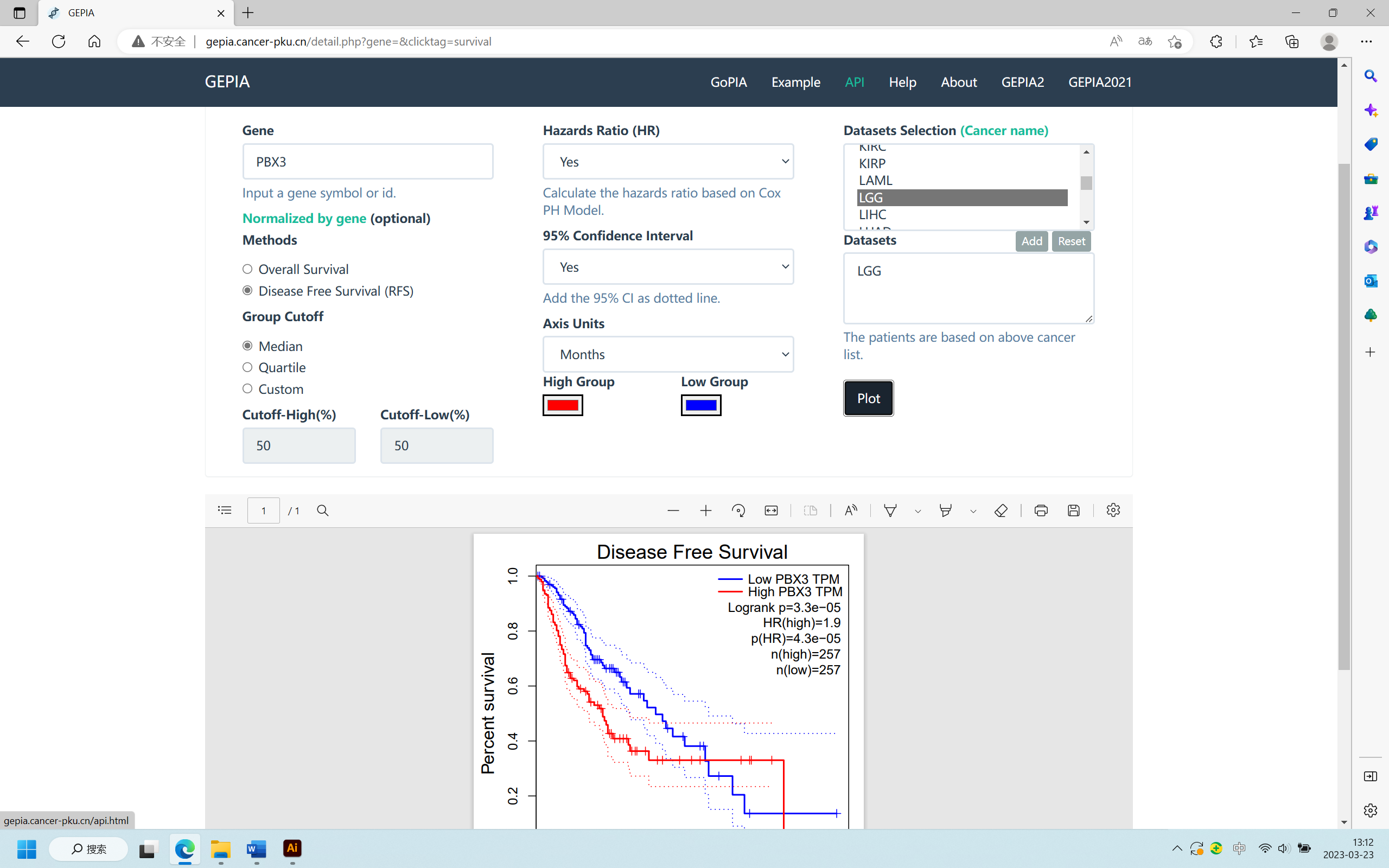


Fig 4E


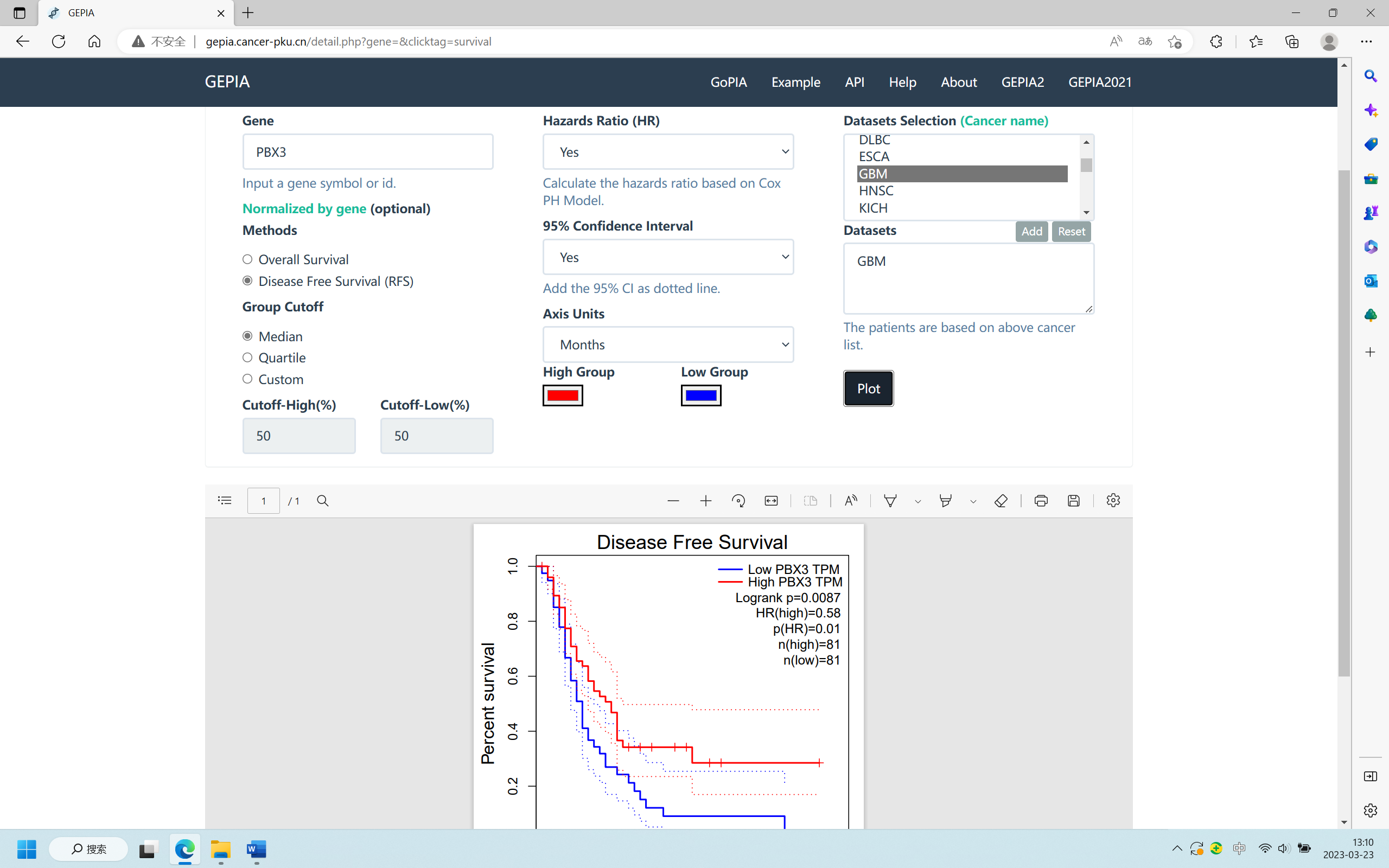

Supplement: S4 Fig — (A), full-grade gliomas according to TCGA databases;(B and C), LGG according to TCGA and GEPIA databases;(D and E), GBM according to TCGA and GEPIA databases. (ZIP) [file pone.0293647.s004.zip › FIG 4/Fig 4Cand E.docx]
