# Supplementary material for: PBX3 as a biomarker for the early diagnosis and prediction of prognosis of glioma
Source: PLoS One. 2024 Feb 7;19(2):e0293647. doi: 10.1371/journal.pone.0293647 (PMC10849273; doi:10.1371/journal.pone.0293647)
Supplement: Attachment — Submitted filename: Response to Reviewers.docx [file pone.0293647.s015.docx]

Response to Reviewers

Thank you very much for the reviewers who raised the valuable comments on this article. I will answer the questions raised by the reviewers.

Response to Reviewer #3:

1 Abbreviation should be used at first use and followed thereafter throughout the manuscript. E.g. LGG, GBM, TCGA, GEPIA, CGGA, CCLE, etc,. Therefore, it is recommended to provide a list of abbreviations.

Response:Thank you for your feedback and suggestions. We appreciate your guidance regarding the inclusion of an abbreviation list. We have taken your suggestion into consideration, and we have added an abbreviation list at the end of the main text of the manuscript.We believe that this addition will enhance the readability and understanding of the manuscript for the readers. Thank you once again for bringing this to our attention, and we hope that this revision meets your expectations.

2 Authors proposed PBX3 as a diagnostic marker, However, it is a transcription factor and may not be present in the biological fluids freely. Therefore, It is important to discuss how PBX3 can be used as a diagnostic marker?

# Response: Thank you for your valuable comments and suggestions. We appreciate your insight regarding the proposed diagnostic marker PBX3.Indeed, PBX3 is known to be a transcription factor, primarily involved in regulating gene expression. We acknowledge that the expression of PBX3 in bodily fluids is not easily detectable. However, existing literature has reported on the regulatory mechanisms of PBX3 expression, which provide evidence for its diagnostic value. Such as, [Hongru Sun](https://pubmed.ncbi.nlm.nih.gov/?term=Sun+H&cauthor_id=31140752)^[1]^ et al confirmed that PBX3 hypermethylation in peripheral blood leukocytes predicts better prognosis in colorectal cancer. In addition,we will further explore the possibility of indirectly assessing PBX3 activity or downstream effects in biological samples, such as measuring its target genes or associated signaling pathways. Additionally, we will emphasize the importance of considering alternative methods or strategies for evaluating PBX3's diagnostic utility, including tissue-based assessments or other complementary biomarkers.We thank you again for bringing up this important point, and we will revise our manuscript accordingly to provide a more comprehensive discussion on how PBX3 can be utilized as a diagnostic marker

References

1 [Hongru Sun](https://pubmed.ncbi.nlm.nih.gov/?term=Sun+H&cauthor_id=31140752) , [Hao Huang](https://pubmed.ncbi.nlm.nih.gov/?term=Huang+H&cauthor_id=31140752) , [Dapeng Li](https://pubmed.ncbi.nlm.nih.gov/?term=Li+D&cauthor_id=31140752) , [Lei Zhang](https://pubmed.ncbi.nlm.nih.gov/?term=Zhang+L&cauthor_id=31140752) , [Yuanyuan Zhang](https://pubmed.ncbi.nlm.nih.gov/?term=Zhang+Y&cauthor_id=31140752),  [Jing Xu](https://pubmed.ncbi.nlm.nih.gov/?term=Xu+J&cauthor_id=31140752) , [Ying Liu](https://pubmed.ncbi.nlm.nih.gov/?term=Liu+Y&cauthor_id=31140752) , [Yupeng Liu](https://pubmed.ncbi.nlm.nih.gov/?term=Liu+Y&cauthor_id=31140752) , [Yashuang Zhao](https://pubmed.ncbi.nlm.nih.gov/?term=Zhao+Y&cauthor_id=31140752). PBX3 hypermethylation in peripheral blood leukocytes predicts better prognosis in colorectal cancer: A propensity score analysis. Cancer Med.2019 Jul;8(8):4001-11.
